# Periostin Facilitates the Epithelial-Mesenchymal Transition of Endometrial Epithelial Cells through ILK-Akt Signaling Pathway

**DOI:** 10.1155/2016/9842619

**Published:** 2016-03-13

**Authors:** Qiao-mei Zheng, Jing-jing Lu, Jing Zhao, Xuan Wei, Lu Wang, Pei-shu Liu

**Affiliations:** Department of Obstetrics and Gynecology, Qilu Hospital of Shandong University, 107 Wenhua Xi Road, Jinan, Shandong 250012, China

## Abstract

Although periostin was confirmed to facilitate the pathogenesis of endometriosis by enhancing the migration, invasion, and adhesion of human endometrial stromal cells (ESCs), its effect on the endometrial epithelial cells (EECs) is still unknown. The current study aimed to determine whether periostin enhanced the epithelial-mesenchymal transition (EMT) of EECs. EECs were isolated from 12 women with endometriosis. The migration and invasion abilities of EECs were evaluated by transwell assays. Expressions of proteins were detected by western blot. After treatment with periostin, the migration and invasion abilities of EECs were enhanced. Additionally, E-cadherin and keratin were downregulated while N-cadherin and vimentin were upregulated in EECs. Simultaneously, levels of ILK, p-Akt, slug, and Zeb1 were all upregulated in EECs. After silencing the expression of ILK in EECs, levels of p-Akt, slug, Zeb1, N-cadherin, and vimentin were downregulated while E-cadherin and keratin were upregulated. Although periostin weakened the above effects in EECs after silencing the expression of ILK, it failed to induce the EMT of EECs. Thus, periostin enhanced invasion and migration abilities of EECs and facilitated the EMT of EECs through ILK-Akt signaling pathway. Playing a pivotal role in the pathogenesis of endometriosis, periostin may be a new clinical therapy target for endometriosis.

## 1. Introduction

Endometriosis, defined as the presence of endometrial and stromal cells at extrauterine locations, is a persistent gynecological problem which can result in dysmenorrhea, infertility, and decreased quality of life [1]. It affects about 10% of women of reproductive age [2]. Nearly two-thirds of adolescents with dysmenorrhea or chronic pelvic pain have laparoscopic evidence of endometriosis [3]. However, there is no radical cure other than surgery for endometriosis due to its unclear pathogenesis. To date, the most widely accepted theory is the retrograde reflux hypothesis, which suggests that endometrial tissues can regurgitate into pelvic cavity during menstruation and develop into endometriosis [4]. However, it is known that cells will die when they detach from extracellular matrix (ECM) or adhere to inappropriate location, namely, anoikis [5]. Epithelial-mesenchymal transition (EMT), a main feature associated with anoikis resistance, plays vital roles in tumor progression and metastatic colonization [6].

EMT is a crucial event in embryogenesis and tumor metastasis characterized by epithelial cells losing epithelial markers and acquiring mesenchymal markers. During EMT, epithelial cells lose cell polarity and are converted into mesenchymal cells, endowing cells with invasive and metastatic properties. Downregulation of E-cadherin, a cell adhesion molecule expressed in epithelial cells, is a crucial molecular feature of EMT [7]. Transcription factors, including snail, slug, Zeb1, and Twist, can initiate EMT via repressing the expression of E-cadherin [8, 9]. Previous studies point out that EMT plays essential roles in the metastasis of tumors [10–12]. Although endometriosis is a benign disease, it behaves malignantly by penetrating and developing elsewhere like cancer metastasis. Additionally, emerging evidences indicate that EMT plays a significant part in the initial formation of endometriosis [13, 14].

Periostin is a secretory extracellular matrix protein which is widely expressed in bone, tooth, heart, uterus, and breast. Abnormally high levels of periostin have been reported in breast cancer, ovarian cancer, and hepatocellular carcinoma [15–17]. Existing evidence suggests that periostin can facilitate tumor metastasis through inducing EMT [18, 19]. As a ligand for integrins, periostin mainly promoted cancer cells invasion and metastasis via integrin pathways [20, 21]. Integrin-linked kinase (ILK), a key role in the integrin pathway, directly phosphorylated its downstream target, such as Akt, resulting in the EMT process [22].

In our previous study, we observed significantly higher expression of periostin in the ectopic and eutopic endometrium of endometriosis [23]. Additionally, we demonstrated that periostin facilitated endometriosis by enhancing the migration, adhesion, and invasion of endometrial stromal cells (ESCs) [24]. But the effect of periostin on the EECs is still unknown. Given that endometriosis behaves malignantly by penetrating and developing elsewhere like tumor metastasis, we herein hypothesize that periostin may facilitate endometriosis by inducing the EMT of EECs. The current study was undertaken to determine whether periostin enhances the EMT of EECs, as well as exploring the mechanism through which periostin favored EMT in EECs.

## 2. Materials and Methods

### 2.1. Sample Collection and Cell Culture

Eutopic endometrium tissues were obtained from 12 women (23–41 years old; menstrual cycle: proliferative phase) with endometriosis. All of the participants were at reproductive age, had regular menstruation, and received no hormonal therapy at least 6 months before the study. Endometriosis was visually diagnosed during the laparoscopy for ovarian cysts and then ascertained by pathological examination. All the participants were from the Department of Obstetrics and Gynecology, Qilu Hospital of Shandong University from July 2014 to May 2015. Informed consent was obtained from all participants prior to surgery. The Institutional Review Board of Shandong University approved the study.

After collection, tissues were immediately washed with PBS to remove blood, mucous, and debris. The EECs were isolated following the digestion of type IV collagenase (5 mg/mL). Cell suspension was filtrated through a sterile stainless steel wire mesh (100 *μ*m) to remove undigested tissues. ESCs were removed by passing a 40 *μ*m sieve. Then, the 40 *μ*m mesh was washed thoroughly upside down with medium to get the EECs. The medium was collected and centrifuged at 1000 rpm for 7 minutes. After removing the supernatant, cells were then cultured at 37°C and 5% carbon dioxide in Dulbecco modified Eagle medium F-12 (DMEM/F12; Sigma-Aldrich, St. Louis, Missouri) containing 10% fetal bovine serum (FBS; Gibco, Australia) and 1% antibiotic. The purity of EECs was evaluated by cell immunofluorescence using mouse antihuman keratin (1 : 50; Cell Signaling Technology, Danvers, Massachusetts) and rabbit antihuman vimentin (1 : 50; Cell Signaling Technology). The purity of EECs was over 95%, which was shown as the proportion of epithelial cells in 5 randomly selected pictures (200x magnification). These cells were used for the following experiments.

### 2.2. Transwell Migration and Invasion Assays

Cells were treated with periostin (20 ng/mL and 40 ng/mL) when reaching 80% to 90% confluence. After treatment for 48 h, cells were digested for migration and invasion assays as previously described [25]. Pictures of stained cells were taken by the Olympus IX51 inverted microscope. Cells were counted in five random fields (200x) of each chamber. The average cell numbers of three duplicate assays for each experimental condition were used for statistical analysis.

### 2.3. Silencing of the ILK Gene in EECs

The small-interfering RNA sequences targeting human ILK (siRNA-ILK) were designed by GenePharma Company (Shanghai, China). Cells were seeded in 6-well plate without antibiotics treated for 12 hours and transfected with blank sequence or siRNA-ILK (50 nmol/L) using lipofectamine 2000 (Invitrogen Life Technologies) when cell confluence gets 50% to 60%. After 48 hours of transfection, cells were digested for the ensuing cell experiments. The siRNA-ILK sequences are listed in Table 1.

### 2.4. Western Blot

Total protein was extracted from samples for western blot, as described previously [25]. Primary antibodies used for immunodetection were anti-ILK, anti-p-Akt, anti-E-cadherin, anti-N-cadherin, anti-vimentin, anti-keratin, anti-slug, and anti-Zeb1 as well as anti-glyceraldehyde-3-phosphate dehydrogenase (GAPDH; Cell Signaling Technology, Danvers, Massachusetts, USA). Secondary antibodies were anti-rabbit and anti-mouse IgG peroxidase conjugate (Zhongshan Jinqiao Biotechnology Co., Ltd., Beijing, China). GAPDH was used as a loading control. The results were quantified by densitometry, using ImageJ software (NIH, Bethesda, MD, USA).

### 2.5. Statistical Analysis

GraphPad Prism Version 5.01 (GraphPad Software, San Diego, California, USA) was used for statistical analysis. Data were shown as mean ± SEM. Student's *t*-test and one-way ANOVA analysis were, respectively, conducted to analyze the differences between groups and among groups. *P* value < 0.05 was considered statistically significant.

## 3. Results

### 3.1. The Purity of EECs

**Figure 1:**
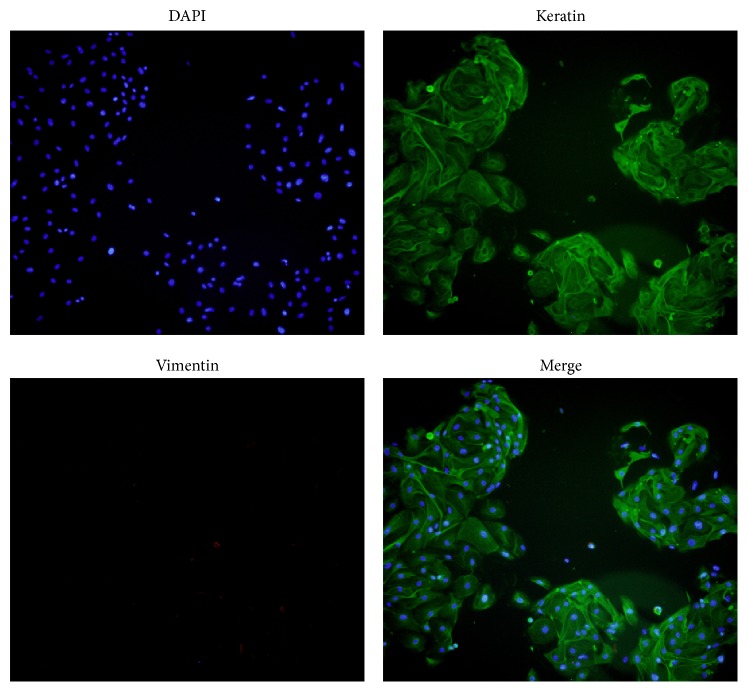
Identification of purity of EECs. Representative staining of cell nucleus is shown in the first image; green immunofluorescence shows the expression of cytokeratin; lack of red fluorescence represents negative immunoreactivity for vimentin; first three images are merged together, as shown in the last picture.

The purity of EECs was 95.6%  ±  3.5%, as confirmed by cell immunofluorescence, which was judged by blue fluorescence for cell nucleus, green fluorescence for keratin, and red fluorescence for vimentin (Figure 1).

### 3.2. Periostin Enhanced the Migration and Invasion Abilities of EECs

**Figure 2:**
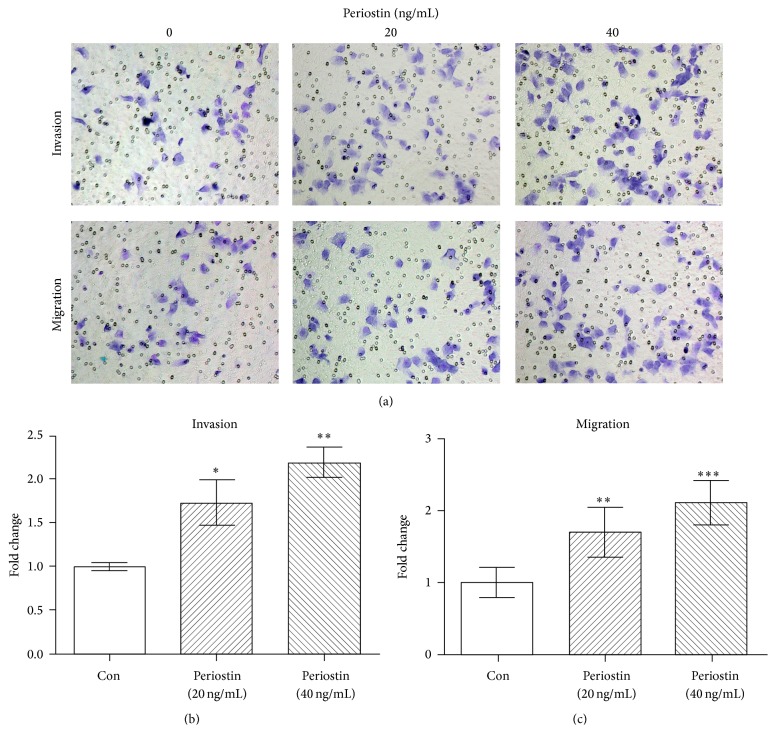
The influence of periostin on the migration and invasion abilities of EECs. ^*∗*^ *P* < 0.05, ^*∗∗*^ *P* < 0.005, and ^*∗∗∗*^ *P* < 0.001.

In our previous study, we detected the concentration of periostin in peritoneal washing fluids of patients with and without endometriosis, which was 48.32 ng/mL and 22.29 ng/mL, respectively (data not published yet). After being treated with periostin (20 ng/mL and 40 ng/mL, resp.) for 48 h, cells were digested for migration and invasion assays. As shown in Figure 2, the migration and invasion abilities of EECs were enhanced by the treatment with periostin, especially for the treatment with 40 ng/mL periostin.

### 3.3. Periostin Facilitated the EMT and Upregulated the Expression of ILK and p-Akt in EECs

**Figure 3:**
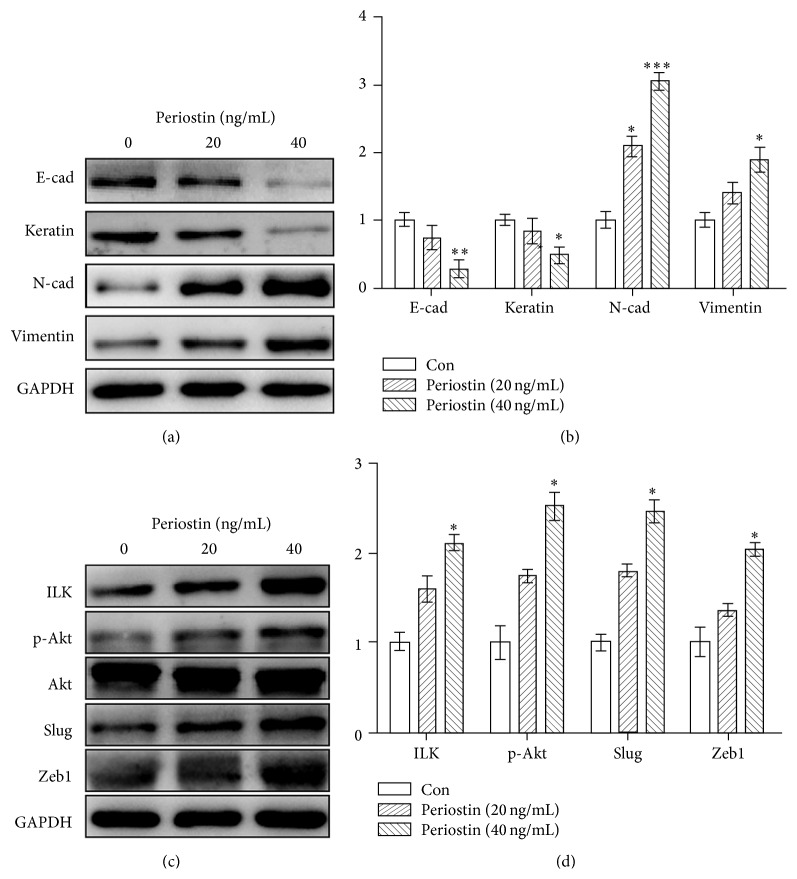
Effects of periostin on EMT-related markers, ILK, and p-Akt in EECs. ^*∗*^ *P* < 0.05, ^*∗∗*^ *P* < 0.005, and ^*∗∗∗*^ *P* < 0.001.

To investigate whether periostin facilitated the EMT of EECs, hallmarks of EMT (E-cadherin, N-cadherin, keratin, and vimentin) were detected by western blot. As depicted in Figures 3(a) and 3(b), the levels of E-cadherin and keratin were decreased in EECs when treated with periostin, though only the 40 ng/mL treatment was statistically significant. On the contrary, the N-cadherin and vimentin expression were markedly increased in the EECs, particularly in the 40 ng/mL treatment. Furthermore, the ILK expression and p-Akt expression were both upregulated by periostin in EECs (Figures 3(c) and 3(d)). As critical transcription factors of EMT, levels of slug and Zeb1 were also markedly upregulated by periostin in EECs (Figures 3(c) and 3(d)). However, only the 40 ng/mL periostin group was statistically significant.

### 3.4. Periostin Facilitated the EMT of EECs through the ILK-Akt Pathway

**Figure 4:**
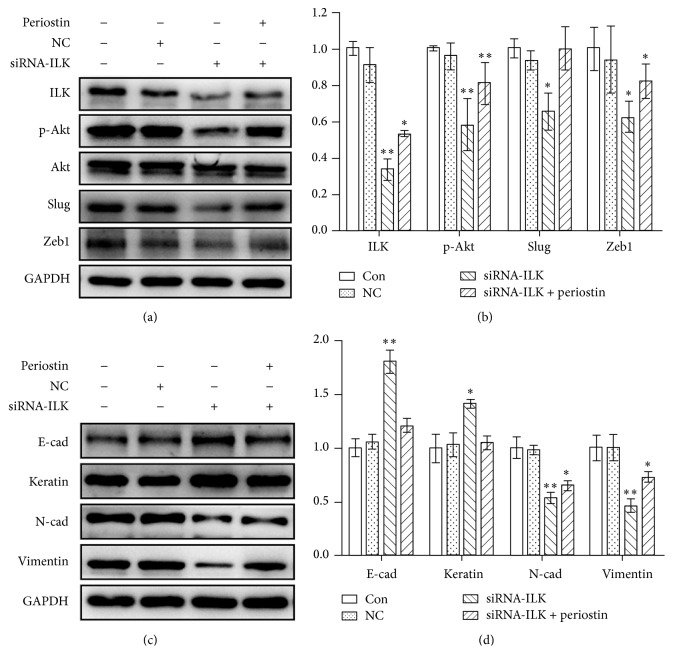
Effects of siRNA-ILK and periostin on ILK, p-Akt, and EMT-related markers in EECs. ^*∗*^ *P* < 0.05, ^*∗∗*^ *P* < 0.005, and ^*∗∗∗*^ *P* < 0.001.

The expression of ILK was obviously decreased after the transfection of 3 siRNA-ILKs in EECs, especially the transfection of ILK-homo-755. So ILK-homo-755 was used in the remaining tests. After being transfected with the siRNA-ILK, ILK, p-Akt, slug, and Zeb1 expressions were all downregulated significantly in EECs, which can be weakened by the addition of periostin (40 ng/mL) (Figures 4(a) and 4(b)). Additionally, E-cadherin and keratin were upregulated while N-cadherin and vimentin were downregulated in EECs after receiving ILK silencing, which can also be weakened by the addition of periostin (Figures 4(c) and 4(d)). Although the above effects were weakened by periostin, periostin failed to induce the EMT of EECs receiving ILK silencing when compared to normal EECs or EECs receiving blank siRNA sequence (Figure 4). Thus, periostin facilitated the EMT of EECs through the ILK-Akt pathway.

## 4. Discussion

Emerging evidences suggest that periostin is overexpressed in various types of human cancers and further results in accelerating migration and invasion abilities of tumor cells [26]. Although endometriosis shares many characteristics with tumors, little is known about the role of periostin in endometriosis. Our previous study showed significantly higher expression of periostin in the eutopic and ectopic endometrium of endometriosis [23]. In line with this, we demonstrated that periostin facilitated the progress of endometriosis by enhancing the adhesion, migration, and invasion of ESCs [24]. However, the effect of periostin on the EECs of endometriosis is still unknown. In the present study, we assessed the effect of periostin on the migration and invasion abilities of EECs of endometriosis and demonstrated that periostin indeed enhanced the migration and invasion abilities of EECs.

Periostin was shown to be not only a new marker of EMT but also an inducer of this program [27]. Hu et al. pointed out that periostin was an important mediator of TGF-*β*-induced EMT in prostate cancer cells and its overexpression promoted cell proliferation, invasion, and migration of prostate cancer [28]. Upregulated periostin significantly promoted the EMT of adamantinomatous craniopharyngioma cells by activating Akt signaling pathway [29]. Silencing of periostin inhibited nicotine-mediated cell growth and EMT in lung cancer cells [30]. Apart from tumors, EMT also plays a significant part in the initial formation of endometriosis [13, 14]. Thus, we propose that periostin may facilitate the EMT of EECs in the pathogenesis of endometriosis. As expected, E-cadherin and keratin were downregulated while N-cadherin and vimentin were upregulated in EECs after being treated with periostin, indicating that periostin induced the EMT of EECs. The results were in line with previous evidences that E-cadherin was downregulated, while N-cadherin and vimentin were upregulated in endometriosis [31–33]. EMT-inducing transcription factors (EMT-TFs) are reported to prominently initiate the EMT by repressing the expression of E-cadherin directly or indirectly [8]. Periostin upregulated snail expression in prostate cancer cells but downregulated Twist expression in bladder cancer cells [18]. Silencing of periostin decreased cell invasion and snail expression in lung cancer cells [30]. So, we examined the effect of periostin on the expression of EMT-TFs in EECs and verified that levels of slug and Zeb1 were both increased after the addition of periostin. Therefore, periostin played crucial roles in the pathogenesis of endometriosis by facilitating the EMT of EECs.

Accumulating evidences suggest that periostin is a ligand for integrin and mainly facilitates the EMT process via integrin pathway [34, 35]. Yan and Shao demonstrated that transduction of periostin into nonmetastatic 293T cells induced cell invasion and metastasis via EMT and the role of periostin in EMT required integrin signaling pathway [19]. ILK, an essential role in the integrin pathway, is a serine-threonine kinase that can directly phosphorylate its downstream targets to mediate cell-ECM and intracellular processes [36]. Besides that, ILK promotes the migration and invasion of cancer cells by facilitating the EMT process [37, 38]. In hepatocellular carcinoma, ILK activated Akt through phosphorylating Akt at Ser473, resulting in EMT of liver epithelial cells and radioresistance and chemoresistance of hepatocellular carcinoma cells [22]. Silencing of the ILK decreased the phosphorylation of Akt and prevented the migration of thyroid cancer cells [39]. Additionally, periostin overexpression induced the EMT of adamantinomatous craniopharyngioma cells by activating Akt signaling pathway [29]. Consistent with this, our previous study demonstrated that periostin enhanced the adhesion, migration, and invasion of ESCs through ILK-Akt pathway in endometriosis [24]. In the present study, the ILK-Akt pathway was activated in EECs after the treatment with periostin. Silencing the expression of ILK decreased the phosphorylation rate of Akt in the EECs, leading to upregulation of epithelial markers of EECs. Moreover, periostin failed to induce the EMT of EECs after silencing the expression of ILK. From the above, ILK-Akt pathway was activated in EECs and periostin facilitated the EMT of EECs through the ILK-Akt pathway.

Recently, studies have been focused on the therapeutic potential by targeting periostin in different diseases. In glioma, Let-7f inhibited the cell proliferation, migration, and invasion by repressing the expression of periostin [40]. Quercetin suppressed the production and function of periostin in human nasal epithelial cells and resulted in improvement of clinical conditions of allergic rhinitis [41]. Given its critical role in the pathogenesis of endometriosis, periostin may be a promising therapy target for endometriosis. Drugs repressing the expression or inhibiting the function of periostin may suppress the progress and recurrence of endometriosis. However, the therapeutic effect of targeting periostin on endometriosis remains to be investigated in the future.

## 5. Conclusion

Our results suggest that periostin facilitated the EMT of EECs through ILK-Akt pathways. Playing critically key roles in the EMT process during the pathogenesis of endometriosis, periostin may be a new clinical therapy target for endometriosis.

## Figures and Tables

**Table 1:** The sequences of siRNA-ILK.

Number	Sense (5′-3′)	Antisense (5′-3′)
ILK-homo-412	UGG ACA CCG UGA UAU UGU ATT	UAC AAU AUC ACG GUG UCC ATT
ILK-homo-755	CAG CUU AAC UUC CUG ACG ATT	UCG UCA GGA AGU UAA GCU GTT
ILK-homo-1486	GAC CCA AAU UUG ACA UGA UTT	AUC AUG UCA AAU UUG GGU CTT
Negative control	UUC UCC GAA CGU GUC ACG UTT	ACG UGA C AC GUU CGG AGA ATT
